# Identification of a pathogenic mutation in *ATP2A1* via in silico analysis of exome data for cryptic aberrant splice sites

**DOI:** 10.1002/mgg3.552

**Published:** 2019-01-28

**Authors:** Christine C. Bruels, Chengcheng Li, Tonatiuh Mendoza, Jamillah Khan, Hemakumar M. Reddy, Elicia A. Estrella, Partha S. Ghosh, Basil T. Darras, Hart G. W. Lidov, Christina A. Pacak, Louis M. Kunkel, François Modave, Isabelle Draper, Peter B. Kang

**Affiliations:** ^1^ Division of Pediatric Neurology, Department of Pediatrics University of Florida College of Medicine Gainesville Florida; ^2^ Department of Health Outcomes & Biomedical Informatics University of Florida College of Medicine Gainesville Florida; ^3^ Division of Genetics & Genomics Boston Children’s Hospital and Harvard Medical School Boston Massachusetts; ^4^ Department of Neurology Boston Children’s Hospital and Harvard Medical School Boston Massachusetts; ^5^ Department of Pathology Boston Children’s Hospital and Harvard Medical School Boston Massachusetts; ^6^ Department of Pediatrics University of Florida College of Medicine Gainesville Florida; ^7^ Molecular Cardiology Research Institute Tufts Medical Center Boston Massachusetts; ^8^ Department of Molecular Genetics & Microbiology University of Florida College of Medicine Gainesville Florida; ^9^ Department of Neurology University of Florida College of Medicine Gainesville Florida; ^10^ Genetics Institute and Myology Institute University of Florida Gainesville Florida; ^11^ Present address: Department of Molecular Biology, Cell Biology and Biochemistry Brown University Providence Rhode Island; ^12^ Present address: Health Sciences Division, Department of Medicine, Center for Health Outcomes and Informatics Research Loyola University Chicago Chicago Illinois

**Keywords:** Aberrant RNA splicing, *ATP2A1*, Brody myopathy, cryptic variants

## Abstract

**Background:**

Pathogenic mutations causing aberrant splicing are often difficult to detect. Standard variant analysis of next‐generation sequence (NGS) data focuses on canonical splice sites. Noncanonical splice sites are more difficult to ascertain.

**Methods:**

We developed a bioinformatics pipeline that screens existing NGS data for potentially aberrant novel essential splice sites (PANESS) and performed a pilot study on a family with a myotonic disorder. Further analyses were performed via qRT‐PCR, immunoblotting, and immunohistochemistry. RNAi knockdown studies were performed in Drosophila to model the gene deficiency.

**Results:**

The PANESS pipeline identified a homozygous *ATP2A1* variant (NC_000016.9:g.28905928G>A; NM_004320.4:c.1287G>A:p.(Glu429=)) that was predicted to cause the omission of exon 11. Aberrant splicing of *ATP2A1* was confirmed via qRT‐PCR, and abnormal expression of the protein product sarcoplasmic/endoplasmic reticulum Ca^++^ ATPase 1 (SERCA1) was demonstrated in quadriceps femoris tissue from the proband. Ubiquitous knockdown of *SERCA* led to lethality in Drosophila, as did knockdown targeting differentiating or fusing myoblasts.

**Conclusions:**

This study confirms the potential of novel in silico algorithms to detect cryptic mutations in existing NGS data; expands the phenotypic spectrum of *ATP2A1* mutations beyond classic Brody myopathy; and suggests that genetic testing of *ATP2A1* should be considered in patients with clinical myotonia.

## 1 INTRODUCTION

Next‐generation sequencing (NGS) technologies are now routinely used to discover pathogenic mutations underlying rare genetic disorders. Unfortunately, in many cohorts, NGS techniques identify pathogenic mutations in a minority of families (Lee et al., 2014; Yang et al., 2014); for neuromuscular disorders, in general, it has been found to be around 40% (Ankala et al., 2015), as well as for limb‐girdle muscular dystrophy in particular (Ghaoui et al., 2015; Reddy et al., 2017, 2016). The genetic etiologies of the remaining families often prove difficult to ascertain, as they may lie in unsequenced regions (for exomes and targeted sequence panels in particular) or may be cryptic mutations in regions that are already sequenced. This is especially true of inherited muscle diseases. Although more than 120 genes have been implicated in these diseases (http://www.musclegenetable.fr/, accessed April 2018) (Bonne, Rivier, & Hamroun, 2017), many affected patients remain undiagnosed despite extensive and costly genetic testing. Recent studies have focused on the identification and characterization of pathogenic mutations in regulatory regions, including canonical RNA splice sites. The majority of in silico tools currently available for identifying RNA splicing effects detect variants only in or near canonical splice sites at intron/exon boundaries (Jian, Boerwinkle, & Liu, 2014) or analyze the effects of individual variants (i.e., Human Splicing Finder (Desmet et al., 2009)) rather than lists of variants in multiple genes. Whole transcriptome sequencing (RNA‐seq) has identified aberrant splicing arising from variants in nonconstitutive splice sites (Cummings et al., 2017); however, this approach requires extraction of high‐quality RNA from specific tissues, which may be difficult to acquire in some cases.

We hypothesize that some families with inherited muscle disease that remain without a genetic diagnosis after standard variant analysis of NGS data may harbor noncanonical splice mutations. We have developed a *Potentially Aberrant Novel Essential Splice Sites *(PANESS) bioinformatics pipeline that can efficiently identify single‐nucleotide variants of interest in existing NGS datasets, including those with specific neighboring sequences. This approach was used to identify a pathogenic homozygous mutation in *ATP2A1* (OMIM #108,730) in a family with a previously undiagnosed neuromuscular disease and may be applied not only to inherited muscle diseases, but also to any Mendelian disease category.

## 2 MATERIALS AND METHODS

### 2.1 Ethical compliance, subject enrollment, and tissue collections

The proband (1406‐1), mother (1406‐2), and father (1406‐3) of a family were enrolled in a genetic study under an institutional research board (IRB) protocol at Boston Children's Hospital. A control subject 1234‐1 was also enrolled under an IRB protocol at the same institution. Saliva samples were collected for genomic DNA extraction from 1406‐1, 1406‐2, and 1406‐3. Primary myoblasts were isolated and cultured under a research protocol from a clinical muscle biopsy that was obtained from subject 1406‐1, and from a muscle biopsy obtained from control subject 1234‐1 (Alexander et al., 2011). An additional young adult female control subject was enrolled at the University of Iowa, and muscle cryosections were obtained from a quadriceps biopsy. Specimens were shared among institutions under an IRB protocol at the University of Florida. Plasmid constructs were generated under the auspices of the Biosafety Program at the University of Florida.

### 2.2 Whole exome sequencing

Whole exome sequencing of DNA samples from 1406‐1, 1406‐2, and 1406‐3 was performed by Claritas Genomics. Briefly, exons were targeted using Ampliseq Exome methodology, and paired‐end sequencing was run on an Ion Proton sequencer (Thermo Fisher Scientific). Data were annotated using Annovar (Wang, Li, & Hakonarson, 2010). Variants were called using Torrent Suite Variant Caller and provided as VCF files.

### 2.3 Standard variant analyses

The SQLite manager add‐in for Firefox (https://github.com/lazierthanthou/sqlite-manager) was used to evaluate variants for potential pathogenicity. Briefly, known single‐nucleotide polymorphisms (SNPs) were considered if the MAF was less than 0.001 in the Exome Aggregation Consortium (ExAC) database (http://exac.broadinstitute.org). Candidate variants were filtered based on inheritance pattern, exonic effect (nonsense, frameshift, essential splice site, or missense variants were considered to be potentially pathogenic), cross‐species conservation, and predicted pathogenicity. Species conservation was determined using the GERP and PhyloP scores from ANNOVAR. Pathogenicity of the variants was predicted using the SIFT, PolyPhen2, LRT, MutationTaster, and FATHMM scores from ANNOVAR. Output from these filtering criteria was used only for screening purposes, and the thresholds used were deliberately liberal. Variant nomenclature follows the recommended HGVS naming conventions (Dunnen et al., 2016).

### 2.4 Development and validation of PANESS pipeline

**Figure 1:**
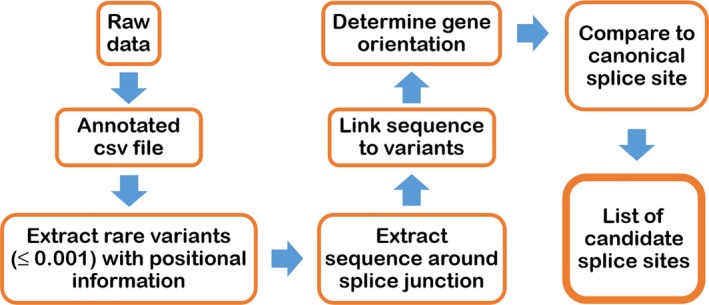
The PANESS pipeline accepts input from a VCF or other variant data file. ANNOVAR is used to determine minor allele frequency (MAF) and gene orientation. Low‐frequency variants (MAF ≤ 0.001) are identified and sequence surrounding each variant is extracted. The definition of donor and acceptor essential splice sites on the appropriate strand is used to determine whether each variant creates a new splice site. Lists of candidate PANESS and the affected genes are created

We developed an in silico tool that evaluates whether variants identified in WES or WGS datasets create novel essential splice sites. The PANESS pipeline (Figure 1) uses SLURM scripts to call existing and custom bioinformatics tools and accepts input from a VCF or other variant data file. ANNOVAR was used to determine MAF and gene orientation (Wang et al., 2010). Low‐frequency variants (MAF ≤ 0.001) and surrounding bases were evaluated for creation of novel GT (donor) or AG (acceptor) dinucleotides. The output included a list of candidate PANESS, their genomic coordinates, the MAF, the affected gene, the type of splice site created, and a comparison of the canonical extended and essential splice site sequence with the equivalent sequence surrounding the PANESS variant. A VCF file (ExAC r0.3.1) was downloaded from the Exome Aggregation Consortium website (Lek et al., 2016) and used to validate performance of the PANESS pipeline. Human Splicing Finder v.3.0 (HSF) (Desmet et al., 2009) (http://www.umd.be/HSF3/) was used to assess the effect on splicing of selected PANESS variants.

### 2.5 Sanger sequencing

Selected PANESS variants in family 1406 were amplified by PCR from genomic DNA to verify cosegregation patterns among affected and unaffected family members using primers flanking the variant of interest. Specifically, the *ATP2A1* (NC_000016.9) variant NC_000016.9:g.28905928G>A; NM_004320.4:c.1287G>A:p.(Glu429=) at the 3’ end of exon 11 was amplified using primers *ATP2A1*_exon10F1: 5'‐TGCCTCCTGAATGAGTTCTCCATC‐3'; and *ATP2A1*_exon12R2: 5'‐GAGTTGCAGGCGTTGGCTCTCTCCAC‐3'. Additionally, cDNA from healthy control myoblasts and from patient‐derived myoblasts was amplified and sequenced to confirm aberrant splicing effects using the primer set above, as well as a second primer pair designed to select for cDNA only (as opposed to gDNA). The forward primer for this second set was located in exon 9 (*ATP2A1*_exon9F1: 5'‐TCTGTTCCGACAAGACAGGC‐3') and the reverse primer spanned the boundary of exons 14 and 15 (*ATP2A1*_ExJ1415R1: 5'‐CGAATGTCAGGTCCGTCTCA‐3'). Sanger sequencing was performed by Eurofins MWG Operon (Hunstville, AL). Sequence data were analyzed using Sequencher v.5.2.3 (GeneCodes Corporation).

### 2.6 Cell culture

Patient‐derived myoblasts were isolated from biopsy tissue obtained from 1406‐1 (proband) and 1234‐1 (control) following a standard protocol (Spinazzola & Gussoni, 2017). The myoblasts were cultured in Skeletal Muscle Cell Growth Medium (PromoCell) with 20% fetal bovine serum (Sigma), 1% GlutaMAX^TM^ (Life Technologies), and 1% Penicillin–Streptomycin (Caisson Laboratories). HEK293 cells were maintained in DMEM (Life Technologies), supplemented with 10% fetal bovine serum. All cell lines were maintained at 37ºC in a humidified incubator supplied with 5% CO_2._

### 2.7 RNA isolation, cDNA generation, and quantitative real‐time PCR

Total RNA was isolated from healthy control and patient‐derived myoblasts using the Zymo Quick‐RNA miniprep kit (Zymogen). RNA was reverse‐transcribed to cDNA using the High‐Capacity RNA‐to‐cDNA Kit (Applied Biosystems). Quantitative real‐time PCR (qRT‐PCR) of cDNA was performed using the TaqMan Gene Expression Master Mix and TaqMan primer probe set designed for *ATP2A1*, with 18S probe sets serving as controls (Thermo Fisher Scientific). Transcript levels were normalized to 18S transcript levels using the ΔΔCT method (Aarskog & Vedeler, 2000). The qRT‐PCR experiment was repeated 3 times.

### 2.8 Protein extraction and immunoblotting

Frozen human muscle samples and whole Drosophila were homogenized in RIPA buffer (25 mMTris‐HCl pH 7.6, 150 mMNaCl, 1% NP‐40, 1% sodium deoxycholate, 0.1% SDS, 1 mM phenylmethylsulfonyl fluoride, 50 mMNaF, 1 mM Na3VO4). After 20 min of centrifugation at 14,000 *g *at 4ºC, the supernatant was collected and protein concentration was determined with the BioRad DC Protein Assay kit. Tissue lysate was diluted with the SDS‐PAGE sample buffer (125 mM Tris‐HCl pH 6.8, 5% 2‐mercaptoethanol, 2% SDS, 10% glycerol, 0.01% bromophenol blue) and heated at 95ºC for 10 min. Equal amounts of total proteins were loaded and resolved on a 4%–12% SDS‐polyacrylamide gel (Life Technologies), then transferred onto a nitrocellulose membrane (20 μm). Primary antibodies used for immunoblotting were as follows: anti‐SERCA1 (1:800, a polyclonal antibody targeting residues surrounding Leu24 near the N terminus, Cell Signaling), anti‐SERCA1 (1:20, Developmental Studies Hybridoma Bank), anti‐GAPDH (1:1,000, Cell Signaling), anti‐β‐actin (1:200, Santa Cruz). For detection, either anti‐mouse peroxidase‐conjugated secondary antibodies or anti‐rabbit peroxidase‐conjugated secondary antibodies (Sigma‐Aldrich) in combination with ECL (Thermo Scientific) were used. Western blot assays were repeated in triplicate.

### 2.9 Immunofluorescence microscopy

Immunofluorescent staining was performed on 10‐μm cryosections of the human muscle samples. Slides were fixed in ice‐cold methanol, washed with phosphate‐buffered saline (PBS), blocked in PBS +5% bovine serum albumin (BSA) for 1 hr at room temperature, then incubated with anti‐laminin (1:100, Abcam), anti‐SERCA1 (1:5, Developmental Studies Hybridoma Bank) overnight at 4ºC. After primary antibody incubation, the slides were washed and incubated with fluorescently labeled secondary antibodies (AlexaFluor^®^ 488 and 594, dilution 1:1,000, Invitrogen) + 1 μg/ml 4′,6‐diamidino‐2‐phenylindole (Sigma‐Aldrich) in PBS +5% BSA for 1 hr at room temperature. After washing in PBS, slides were mounted and immunofluorescence images were acquired.

### 2.10 Vector Constructions

To test the prediction that the variant leads to expression of a truncated protein, the coding region of human *ATP2A1* was amplified by PCR from healthy control and patient‐derived cDNA. Each PCR product was first cloned into the pGEM‐T^®^ vector (Promega). Subcloning of *ATP2A1* cDNA into the pEBG GST fusion vector (Addgene) in‐frame with the GST tag was done by using 5’‐ATTATACTAGTATGGAGGCCGCTCATGCTA‐3’ (forward) and 5’‐AATATGCGGCCGCTTATCCCTCTAGGTAGTT‐3’ (reverse) primers together with Spel/NotI restriction enzyme sites. Generation of the correct clones was confirmed by Sanger sequencing.

### 2.11 Immunoprecipitation and GST Pull‐down assays

HEK293 cells were transfected with each expression plasmid using Lipofectamine 2000 reagent (Life Technologies). At 48 hr post‐transfection, cells were lysed in RIPA buffer and spun at 14,000 *g *at 4ºC for 20 min. Whole cell extracts were immobilized on GST‐beads at 4ºC overnight and eluted from beads by heating in SDS‐PAGE sample buffer. Briefly, as described above, the immunoprecipitants were loaded, resolved, and transferred onto a nitrocellulose membrane. The immunoprecipitants were probed with antibodies to GST at a 1:1,000 dilution (Cell Signaling), incubated with horseradish peroxidase‐conjugated secondary antibodies (Sigma‐Aldrich), and visualized by chemiluminescence. Transfection and GST pull‐down assays were repeated in triplicate.

### 2.12 Drosophila studies

The *UAS‐ds‐SERCA *RNAi lines (genotype: *y^1^ v^1^; P{TRiP.JF01948}attP2*, FBst0025928; and y^1^ sc^*^ v^1^; P{TRiP.HMS02878}attP2, FBst0044581)(Mauvezin, Nagy, Juhasz, & Neufeld, 2015), as well as Gal4 driver lines listed in Table 1, were purchased from the Bloomington *Drosophila* Stock Center (Indiana University, Bloomington, IN). The *TinC‐Gal4* driver line (expression in the heart) was donated by Dr. Matthew J. Wolf (Duke University, Durham, NC). All strains were raised at 25ºC in a 12‐hr light/12‐hr dark cycle on standard *Drosophila* media. To generate flies that downregulate Drosophila *SERCA*, transgenics carrying the *UAS‐ds‐SERCA *RNAi construct were crossed with *Gal4* driver flies at 29°C. For each *Gal4* driver assessed, the number of viable adult progeny observed in each cross ranged from 31 to 120 flies. The negative geotaxis assay was done using *Mhc‐Gal4>UAS‐ds‐SERCA RNAi *flies vs. *CyO; UAS‐ds‐SERCA *control sibling flies (that eclose in the same vial and are raised along RNAi flies). Histological analyses were performed in the Department of Pathology and Laboratory Medicine, Tufts Medical Center. Forty‐day‐old *Mhc‐Gal4>UAS‐ds‐SERCA* RNAi flies and *CyO; UAS‐ds‐SERCA* control sibling flies were immersed in 10% neutral‐buffered formalin (Azer Scientific) for seven days, embedded in paraffin, and sectioned at 5 µm using standard techniques. All sections were stained with hematoxylin and eosin, then examined using standard bright‐field light microscopy. Knockdown of *SERCA* was assessed by immunoblotting as described in 2.8 above.

**Table 1:** Effect of tissue‐specific RNAi‐mediated knockdown of fly *SERCA*. A series of GAL4 drivers was used to target transgene expression in specific tissue/cell types. Lethality is observed when using ubiquitous drivers, or selected muscle drivers

Gal4 driver Symbol	FB stock#	Expression	Myogenesis	Phenotype
P{***Act5C***‐GAL4}25FO1	4414	Ubiquitous	All stages	Lethal^a^
P{GAL4‐***twi***.G}108.4	914	Embryonic and adult muscle precursors	Precursors (high twist inhibits differentiation)	Viable
P{GawB}***kirre***[rP298‐G4]	66682	Muscle progenitors and founders	Fusion	Lethal
P{GawB}***how***[24B]	1767	Mesoderm/all muscles, tendons	Early differentiationMigration/ attachment	Lethal
P{GAL4‐***Mef2***.R}3	27390	All muscles	Differentiation	Lethal (at the L1/ L2 larval stage)
P{***Mhc***‐GAL4.F3‐580}2	38464	All muscles	Differentiated muscles	Locomotor defect
P{GawB}***sr***[md710]	26663	Tendons	Attachment	Viable
***tinC***‐GAL4	Acad.lab	Heart		Viable
P{GawB}***D42***	8816	Motor neurons		Viable
P{***Lsp2***‐GAL4.H}3	6357	Fat body		Viable
P{***drm***‐GAL4.7.1}1.1	7098	Intestinal tract / Malpighian tubules		Viable

a Lethality in the progeny was seen when using either FBst0025928, or FBst0044581, *UAS‐ds‐SERCA* parental line (Material and Methods). Subsequent genetic analyses were done using the FBst0025928 fly line.

### 2.13 Statistical analyses

For qRT‐PCR, western blotting and fly negative geotaxis assay, data were presented as mean ± *SEM* (*n* = 3). Statistical analysis was performed using Sigmaplot 14.0 software. Data were analyzed using one‐way analysis of variance (ANOVA), followed by the Holm–Sidak post hoc test. A *p*‐value ≤0.05 was considered statistically significant.

## 3 RESULTS

### 3.1 Case report

**Figure 2:**
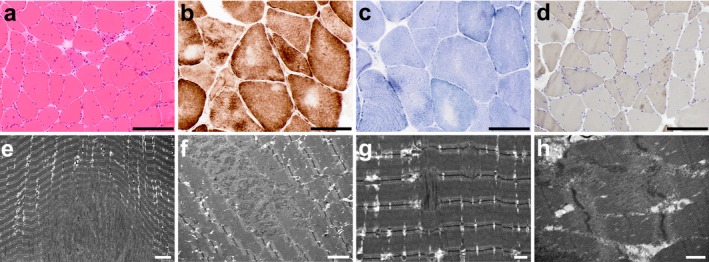
Histological images of quadriceps muscle biopsy on proband 1,406‐1. (a) Hematoxylin and eosin stain shows excessive variation in fiber diameter with both hypertrophic and atrophic fibers and increased centralized nuclei. There is no inflammation and no frank degeneration. There is minimal increased connective tissue. Scale bar, 200 microns. (b) Cytochrome oxidase histochemistry shows a normal stippled pattern, with central pallor suggestive of cores or targets. Scale bar, 100 microns. (c) NADH histochemistry shows a normal stippled pattern, with central pallor suggestive of cores or targets. Scale bar, 100 microns. (d) ATPase stain at pH 9.4 shows mild fiber type grouping of both type 1 and type 2 fibers; the majority of small angulated atrophic fibers are type 2. Scale bar, 200 microns. (e–h) On electron microscopy, the thick and thin filaments, Z lines, M lines, A and I bands are present and generally well‐aligned. Myofiber nuclei, sarcolemmal membranes, and basement membranes are intact. However, there are foci, some quite large, of myofibrillar disorganization and loss of Z‐band structure. Elsewhere, there is streaming of the Z lines and myofibrillar disarray. Nemaline rods are not present, but there is the suggestion that the actin filaments may in some places insert into malformed Z‐band material. There is no increased accumulation of glycogen, abnormal lipid, or other storage material. The mitochondria are normal in number, distribution, and morphology without paracrystalline, osmophilic inclusions, or concentric cristae. There are no vacuolar or degenerative changes in the myofibers

Family 1406 is a consanguineous family from Colombia in which the proband, now a 21‐year‐old young man, was diagnosed clinically with paramyotonia congenita. The proband's mother first sought medical attention for him when he was about a year old as she was concerned about his unusual stiff‐legged gait. His pediatrician did not find any concerning features on his developmental history or examination at that time. As he continued to develop, his parents noticed he would not bend his knees when jumping, and that his gait would sporadically become “stiff legged.” A neurologist found good reflexes and no weakness. His symptoms worsened over time. He tried to be active in soccer and other sports, but he was slower than his peers. He reports no difficulty initiating movements; however, after running for several yards, he experiences a sudden onset of muscle stiffness in his legs that forces him to stop to take a break. After a brief rest, he can then resume running without difficulty. The stiffness is not accompanied by any pain or weakness, but worsens with exposure to cold temperatures. He also has noticed that after gripping a lacrosse or hockey stick for a length of time, he has difficulty releasing the stick. He also reports jaw stiffness with prolonged chewing but has not experienced choking or dysphagia. Neither parent had any muscle symptoms. Physical examination at 16 years showed eyelid, hand, and foot myotonia with mild percussion myotonia at the thenar eminences and the extensor digitorum communis. Physical examination at 20 years showed eyelid myotonia and mild grip myotonia, with no signs of percussion myotonia. Strength was normal in all extremities, with no muscle hypertrophy or atrophy and no heel cord contractures. Electrocardiogram and echocardiogram were both normal. Nerve conduction studies and electromyography (EMG) performed at 12 years was normal. An EMG at 16 years showed normal sensory and motor nerve conduction studies, while on needle examination, fibrillation potentials were noted, without frank myotonic discharges, in the left extensor digitorum communis. Muscle biopsy showed excess in fiber size variability, increased centralized nuclei, mild fiber type grouping, and focal myofibrillar disarray (Figure 2). Clinical genetic testing showed no pathogenic mutations in *CLCN1* and *SCN4A*. Mexiletine did not relieve his symptoms. The clinical impression was that he had an atypical form of paramyotonia congenita.

### 3.2 Genetic analyses, PANESS pipeline, and cosegregation

**Figure 3:**
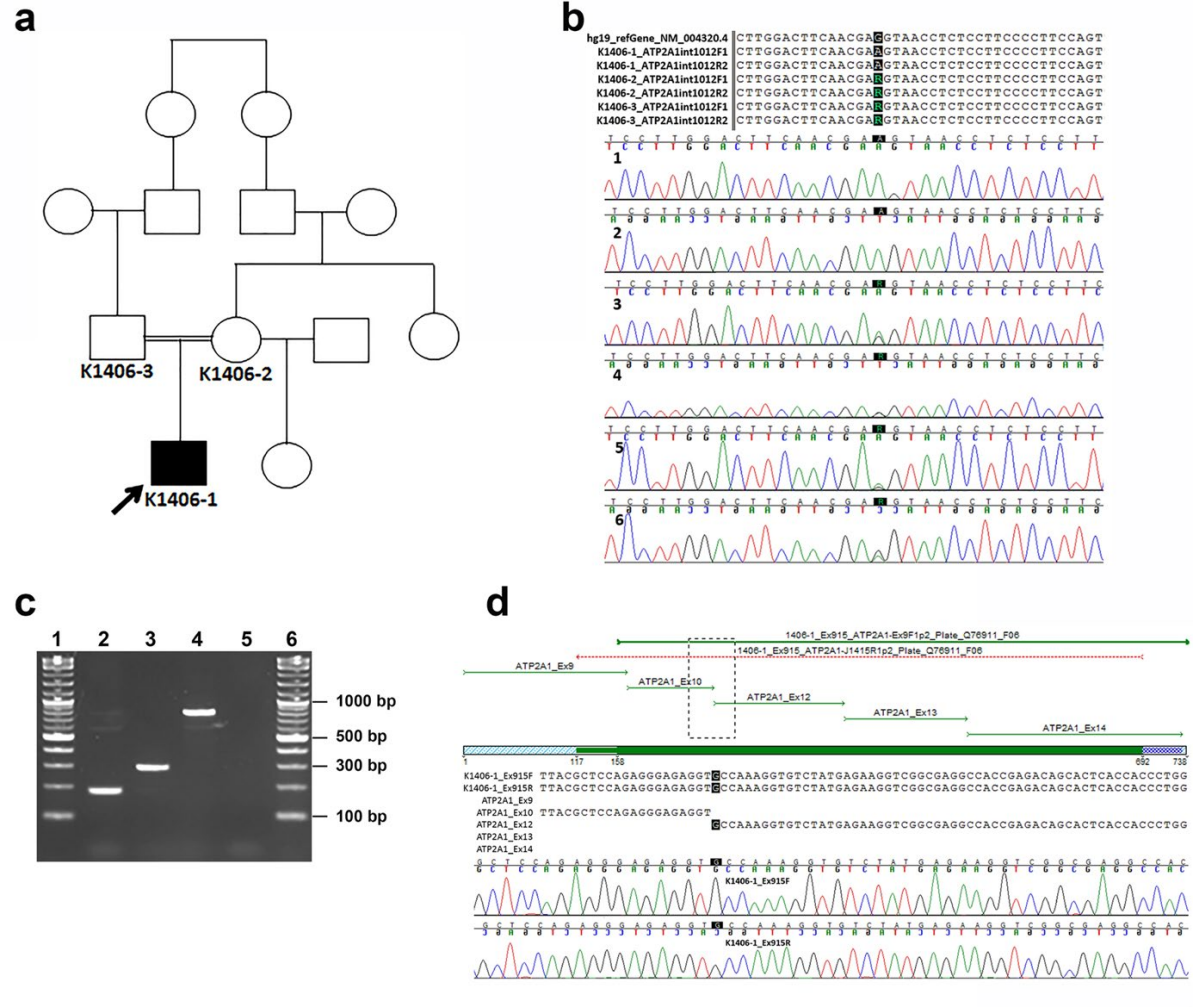
Analysis of family 1406. (a) A family pedigree illustrates the source of consanguinity; the family is from Colombia, and the great‐grandmothers of the proband are sisters. (b) Sanger sequencing of *ATP2A1* (NC_000016.9) confirmed a homozygous *ATP2A1:*c.1287G>A variant (NC_000016.9:g.28905928G>A; NM_004320.4:c.1287G>A:p.(Glu429=)) in 1406‐1 (chromatograms 1 & 2) and a heterozygous *ATP2A1*:c.1287G>A variant in 1406‐2 and 1406‐3 (chromatograms 3–6). (c) Primers *ATP2A1*_exon10F1 and *ATP2A1*_exon12R2 were used to amplify exons 10–12 in cDNA from 1406‐1 (proband) myoblasts (lane 2), along with cDNA (lane 3) and gDNA (lane 4) from healthy control myoblasts. The 1406‐1 amplicon is approximately 100 bp smaller than the control amplicon on a 2% agarose gel. Lanes 1 and 6 show the 2‐log DNA ladder (New England Biolabs), and lane 5 shows a negative control. (d) Sanger sequencing confirmed the omission of exon 11 in cDNA from patient‐derived myoblasts

The great‐grandmothers of the proband were sisters (Figure 3a). Standard variant analysis of exome sequencing data on 1406‐1, 1406‐2, and 1406‐03 showed no obvious pathogenic variants. The PANESS pipeline identified 1,200,951 PANESS variants in the reference ExAC database, and returned a list of the variant information in formats suitable for use in further characterization. For family 1406 in particular, the pipeline identified 16 autosomal recessive PANESS variants, one of which is present as a homozygous variant at the 3’ end of exon 11 of *ATP2A1* (NC_000016.9:g.28905928G>A; NM_004320.4:c.1287G>A:p.(Glu429=)). A review of the initial standard variant analysis showed that this variant was originally annotated as a heterozygous, synonymous variant and thus not considered to be potentially pathogenic. Additionally, this *ATP2A1*:c.1287G>A variant has been reported (rs200596448) as heterozygous in a single individual in both the 1,000 Genomes (Colombian population, accessed April 2018, https://www.ncbi.nlm.nih.gov/variation/tools/1000genomes/) and ExAC (Latino population, Exome Aggregation Consortium Database, https://exac.broadinstitute.org, accessed April 2018) databases. It was also reported as heterozygous in two individuals in the gnomAD database (Latino population, Genome Aggregation Database, https://gnomad.broadinstitute.org, accessed April 2018), with an overall minor allele frequency (MAF) of 8.1e–06. This variant was selected for further analyses since *ATP2A1* is associated with Brody myopathy and is aberrantly spliced in myotonic dystrophy. HSF analysis indicated that this variant was likely to affect splicing through modification of an essential donor site and an exonic splice enhancer site as well as creation of an acceptor splice site.

### 3.3 Confirmation of the variant in gDNA and demonstration of a splicing defect in cDNA

**Figure 4:**
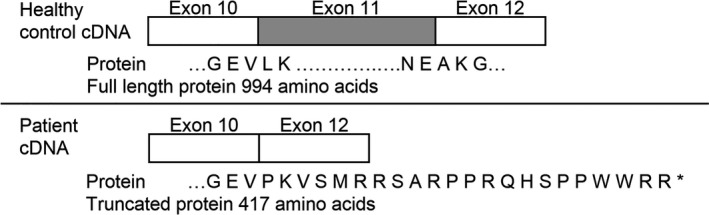
Diagram represents the predicted effect of skipping exon 11 in *ATP2A1* (NC_000016.9) on the SERCA1 amino acid sequence. The variant is the last nucleotide at the 3’ end of exon 11 of *ATP2A1* (NC_000016.9:g.28905928G>A; NM_004320.4:c.1287G>A:p.(Glu429=)). It is predicted to affect splicing through the modification of the canonical donor site (splicing signal decreased by 74%), creation of a cryptic acceptor site (splicing signal increased by 66%), and disruption of an exonic splice enhancer site. The predicted omission of exon 11 was confirmed experimentally at the RNA level. The absence of full length and truncated SERCA1 protein expression in the patient is likely due to nonsense‐mediated decay of the mRNA

Sanger sequencing confirmed that the *ATP2A1*:c.1287G>A variant was present in the homozygous state in 1406‐1 (proband), and present in the heterozygous state in 1406‐2 (mother) and 1406‐3 (father) (Figure 3b). Amplification of cDNA derived from myoblasts cultured from 1406‐1 and a control subject yielded a PCR product approximately 100 bp smaller from 1406‐1 compared to the control (Figure 3c). Sanger sequencing revealed that exon 11 (103 bp) was absent in the 1406‐1 amplicon (Figure 3d) but present in the control cDNA amplicon (data not shown). Skipping of exon 11 is predicted to result in a frame shift and premature stop codon, leading to a truncated protein (p.(Leu396ProfsTer23)) (Figure 4).

### 3.4 *ATP2A1* mRNA expression is decreased in patient‐derived myoblasts

**Figure 5:**
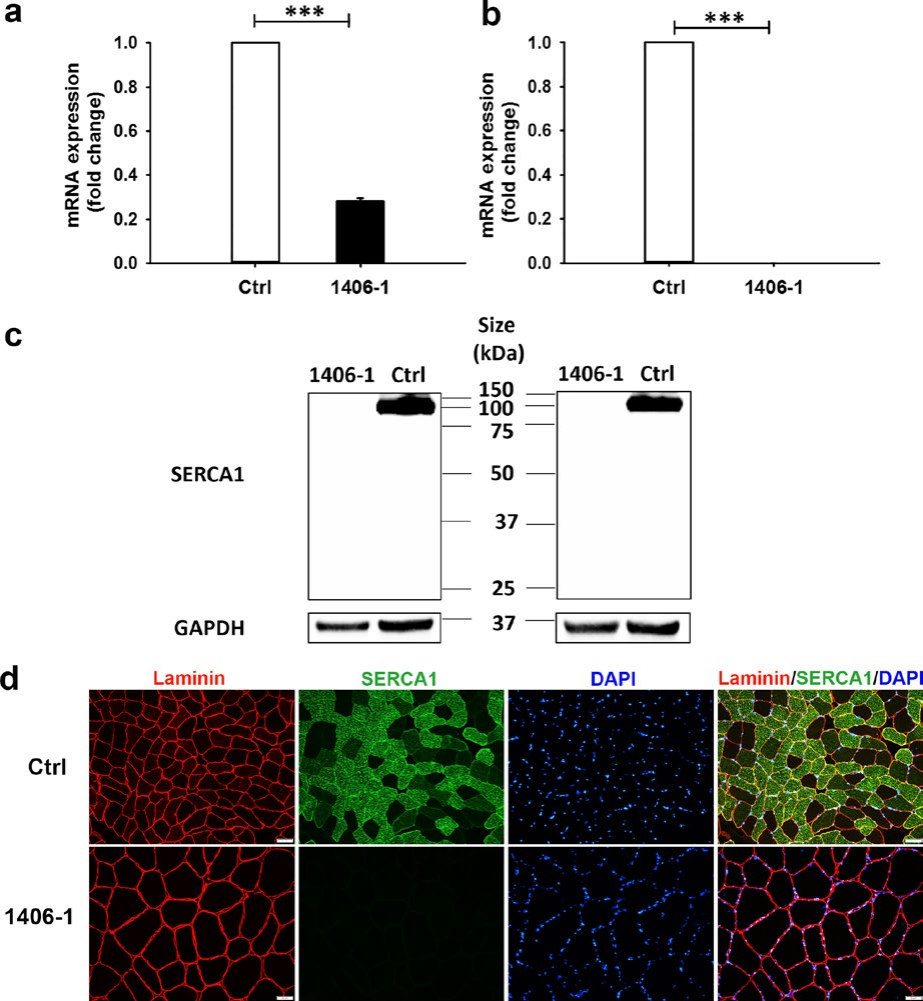
Analysis of mRNA and protein from proband 1406‐1 and a healthy control. (a) qRT‐PCR of cDNA with probes spanning *ATP2A1* (NC_000016.9) exons 9 and 10 show reduced expression of exons 9 and 10 in 1406‐1 compared to control. (b) qRT‐PCR with probes spanning *ATP2A1* exons 11 and 12 confirmed undetectable expression of exons 11 and 12 in 1406‐1 compared to control. (c) Representative western blot of SERCA1 protein in control and 1406‐1 quadriceps femoris muscle. The anti‐SERCA1 antibody (Cell Signaling) targeting an epitope surround Leu24 near the N‐terminus of human SERCA1 detected a band of ~100 kDa in control, but no SERCA1 expression in 1406‐1 (left). The SERCA1 expression pattern was confirmed by another anti‐SERCA1 antibody (Developmental Studies Hybridoma Bank) with the same amount of protein loaded (right). (d) Immunofluorescence analysis of laminin (red) and SERCA1 (green) in quadriceps cross‐section from control and 1406‐1. Nuclei were stained with DAPI. Scale bar: 50 μm. Control muscle tissue displays SERCA1 expression most prominently in fast‐twitch fibers, whereas SERCA1 expression is completely absent in muscle tissue obtained from 1406‐1

The qRT‐PCR using probes spanning exons 9–10 revealed that *ATP2A1* expression in patient‐derived myoblasts was approximately 0.30 (*p* < 0.001) compared to *ATP2A1* expression in healthy control myoblasts (Figure 5a). Additional qRT‐PCR using probes spanning exons 11–12 resulted in undetectable levels of *ATP2A1* expression (*p* < 0.001) in patient‐derived myoblasts compared to expression in healthy control myoblasts (Figure 5b). Moreover, expression of patient‐derived *ATP2A1* cDNA in HEK293 cells results in truncated sarcoplasmic/endoplasmic reticulum Ca^++^ ATPase 1 (SERCA1, Supporting information Figure S1).

### 3.5 Expression of SERCA1 is markedly decreased or absent in patient muscle tissues

SERCA1 expression was compared between control and 1406‐1 quadriceps femoris muscle using western blotting and immunofluorescence staining. Western blotting on muscle lysate samples with two different SERCA1 antibodies showed a significant difference in SERCA1 expression between control and 1406‐1 muscle. Bands consistent with the presence of full‐length SERCA1 (~100 kDa) were seen in protein extracted from control quadriceps; in contrast, no expression of either full length or truncated SERCA1 was found in protein extracted from the 1406‐1 muscle sample using the first anti‐SERCA1 antibody (Cell Signaling, Figure 5c, left). Similar results were found with a different SERCA1 antibody (Developmental Studies Hybridoma Bank, Figure 5c, right). The absence of SERCA1 in 1406‐1 muscle was confirmed by immunohistochemistry, performed with the second anti‐SERCA1 antibody (Developmental Studies Hybridoma Bank) (Figure 5d). In control muscle tissue, SERCA1 was predominantly expressed in fast‐twitch fibers (Figure 5d).

### 3.6 Downregulation of *SERCA* (the Drosophila homolog of *ATP2A1*) at specific stages of myogenesis leads to lethality

**Figure 6:**
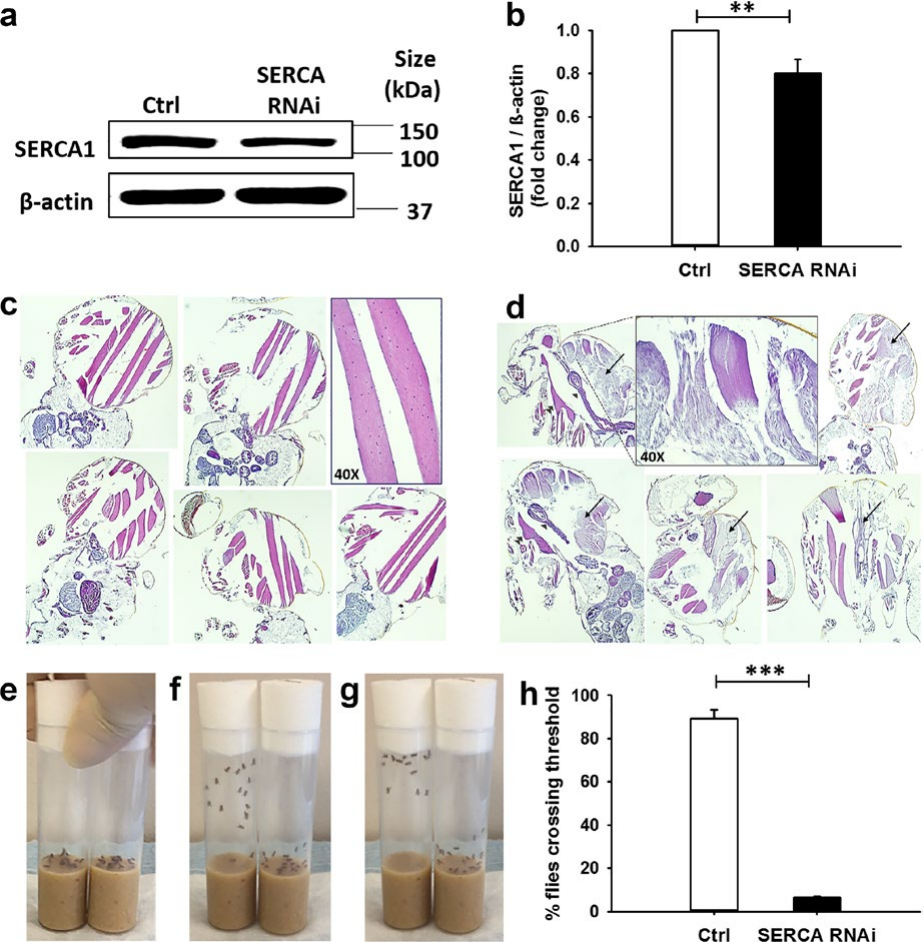
Adult Drosophila that downregulate *SERCA *in differentiated muscles display marked defect in locomotor activity. (a) Representative western blot and (b) relative quantification of SERCA protein from control sibling flies (abbreviated genotype *CyO; UAS‐ds‐SERCA*) and *SERCA* RNAi flies (abbreviated genotype *Mhc‐Gal4; UAS‐ds SERCA*). Western blot showing knockdown of SERCA in whole Drosophila lysates. (c, d) Histological analysis of 40 day‐old *Mhc‐Gal4>UAS‐ds‐SERCA* RNAi flies and *CyO; UAS‐ds‐SERCA* control siblings. (c) None of the control flies showed the muscle defects (*n* = 17). Five representative control flies are shown (10× magnification), as well as magnified muscle fibers (inset, 40×). (d) Severe alterations are seen in the large thoracic muscles of *SERCA* RNAi flies, including the indirect flight muscles and jump muscles (arrows). All *SERCA* RNAi flies but one display widespread muscle breakdown (*n* = 15). Other thoracic organs that are readily visible in some samples appear intact (single arrow head, midgut; double arrow head, ventral nerve cord). Five representative flies are shown (10× magnification), as well as magnified muscle fibers (inset, 40×). (e–h) Adult *SERCA RNAi *and control sibling flies were collected and functionally characterized. (e–g) In each picture, the left vial contains the control flies, and the right vial contains the *SERCA* RNAi flies. (e) The flies were prompted by tapping both vials simultaneously once on a flat surface. (f–g) The climbing ability of the flies was video recorded. Representative still photographs are shown. Most control siblings reach the top in <10 s, while *SERCA *RNAi flies move markedly more slowly. The corresponding video can be accessed in the Supporting information Video S1. (h) Negative geotaxis assay. Control siblings (*n* = 10–14) and *SERCA* RNAi flies (*n* = 9–13) were assessed for their ability to cross a 3‐cm‐threshold line in 12 s. The assay was performed in triplicate and repeated three times at 1‐min intervals (9 times total). Few *SERCA* RNAi flies were able to reach the line in the given time compared to control siblings

Drosophila *SERCA* (aka *Ca‐P60A,* *FBgn0263006*) encodes the sarco/endoplasmic reticulum calcium pump SERCA, which shows 72% identity to the human protein counterpart (DRSC ortholog prediction tool, Harvard Medical School). Utilizing the well‐established *Gal4/UAS* bipartite system (Brand & Perrimon, 1993), we generated and characterized *SERCA* RNAi Drosophila mutants. Flies that downregulated *SERCA* in the entire organism did not reach adulthood. The lethality phenotype was recapitulated when downregulation of *SERCA* was targeted to differentiating, or fusing myoblasts (i.e., *Mef2*, or *kirre*, positive cells, respectively), but not to muscle precursors maintained in the undifferentiated stage (i.e., *twist *positive cells) (Table 1). Expression of *SERCA *RNAi in myosin heavy chain (Mhc)‐positive muscle fibers resulted in adult flies that were viable (*MhcG4>SERCA RNAi* flies). Downregulation of *SERCA* in corresponding RNAi adult flies was confirmed by western blot analysis of protein lysates prepared from the entire organisms (Figure 6a–b; of note, the analysis underestimates the actual knockdown of SERCA in muscles, since nonmuscle tissues express WT levels of the protein). Histological analysis of adult *MhcG4>SERCA* RNAi flies showed pronounced abnormalities in the large thoracic muscles, including extensive myofibrillar disorganization and loss of structure, striation and nuclei (Figure 6c–d). In addition, these flies displayed decreased locomotor activity (Figure 6e–h and Supporting information Video S1). Downregulation of *SERCA* in a panel of other tissues, for example, motor neurons, fat body, or gastrointestinal tract was tolerated (Table 1).

## 4 DISCUSSION

The *ATP2A1*:c.1287G>A variant identified in family 1406 was not identified upon standard variant analyses. It has been reported in the heterozygous state in two individuals of Latino ancestry, in the Genome Aggregation Database (gnomAD) (Lek et al., 2016), the largest public‐access sequence database currently available. It is a synonymous variant, not predicted to be deleterious by PolyPhen2, SIFT, LRT, or Mutation Taster, with a relatively low CADD score of 2.09. However, it was identified as a PANESS variant by our pipeline, and HSF analyses predicted altered splicing through the modification of the canonical donor site (splicing signal decreased by 74%), creation of a cryptic acceptor site (splicing signal increased by 66%), and disruption of an exonic splice enhancer site. The predicted omission of exon 11 due to destruction of the canonical donor site was confirmed experimentally at the mRNA level in the proband, although no effect of the predicted cryptic acceptor site was observed experimentally. The absence of SERCA1 protein expression indicates that the defective mRNA is likely subject to nonsense‐mediated RNA decay; it is less likely that the truncated protein is produced but is unstable and quickly degraded.

The proband in this study has some of the classic features of Brody myopathy, which is characterized by early onset episodes of painless muscle cramps induced by exercise and relieved by brief rest periods, made worse by exposure to cold temperatures (Brody, 1969; Voermans et al., 2012). Brody myopathy is the classic human disease associated with mutations in *ATP2A1* to date (Odermatt et al., 2000, 1996). However, there are some atypical features of the patient reported here. There were clear clinical signs of myotonia, albeit without classic myotonic discharges on electromyography. In addition, electromyography showed subtle features of denervation rather than the silent cramps or myopathic features that have been reported numerous times. The muscle biopsy also showed suggestions of histological features found in some congenital myopathies (Jungbluth et al., 2018). These findings indicate that Brody myopathy, along with genetic analysis of *ATP2A1*, should be considered in patients with clinical myotonia of unclear etiology, as well as patients who have findings suggestive of congenital myopathy on muscle biopsy. The range of phenotypes with *ATP2A1* mutations also includes malignant hyperthermia (Sambuughin et al., 2014).

The protein encoded by *ATP2A1*, SERCA1, is a pump expressed in type 2 (fast twitch) skeletal muscle that catalyzes the ATP‐dependent transport of Ca^++^ from the sarcoplasm into the sarcoplasmic reticulum. SERCA1 has four transmembrane helices that are involved in Ca^++^ binding and translocation, as well as ATP binding and phosphorylation domains in the cytoplasmic portion of the protein (MacLennan, Rice, & Odermatt, 1997). A transcript variant (S1T) that skips exon 11 was previously described (Chami et al., 2001). Skipping of exon 11 in S1T creates a frameshift with a premature stop codon in exon 12. The expressed S1T protein lacks three of the transmembrane helices involved in Ca^++^ binding, as well as the cytoplasmic ATP binding and phosphorylation domains. qRT‐PCR analyses showed differential S1T expression in human adult and fetal tissues, with S1T not expressed in adult or fetal heart or skeletal muscle, nor in adult brain. In vitro studies in multiple human cell lines showed that S1T expression disrupted normal Ca^++^ regulation. Additionally, patients with Brody myopathy have impaired sarcoplasmic reticulum Ca^++^ ATPase activity (MacLennan, 2000). Thus, mutations in *ATP2A1* lead to abnormalities of Ca^++^ transmembrane flux that help to explain the stiffness and clinical myotonia in our patient. Of note, *ATP2A1* is one of the multiple RNA targets that are misspliced as a result of MBNL1 sequestration in myotonic dystrophy (DM) muscles (Hino et al., 2007; Kimura et al., 2005). Our report further highlights the potential contribution of this gene, together with *CLCN1*, to the mechanism of the myotonia shown by DM patients. Type II fibers predominate in mouse diaphragm, and SERCA1‐null mice have been observed to die from respiratory failure shortly after birth (Pan et al., 2003). In Drosophila, previous studies have shown that missplicing of *SERCA *leads to hypercontraction in flies (a phenotype which is correlated with myotonia) (Picchio et al., 2018). Fly SERCA had also been demonstrated to play an important role in mediating calcium storage‐regulation of muscle action potential (Sanyal et al., 2005), and cardiac function (Abraham & Wolf, 2013; Sanyal, Jennings, Dowse, & Ramaswami, 2006). The current studies suggest that selected stages of myogenesis may be differentially sensitive to decreased levels of this conserved ATPase.

Our PANESS pipeline is powerful with respect to aberrant splice site detection, but there are numerous other types of noncoding sequence variants that may cause disease as well. We plan to develop more sophisticated algorithms that can evaluate extended splice sites, which have more variability than essential splice sites. A recently published bioinformatic tool, CryptSplice (Lee et al., 2017), uses machine learning to identify and evaluate the potential pathogenicity of splice variants, which we may incorporate in future iterations of the PANESS pipeline. Additionally, identification of a splice defect caused by a variant that was initially assessed as nonpathogenic by standard NGS variant analysis highlights the need for multiple approaches for in silico variant discovery when next‐generation sequencing data exist and appear to be nondiagnostic based on standard analyses, whether in a clinical genetic laboratory or in a research setting. However, the PANESS pipeline is intended to be used for research purposes to interrogate existing NGS databases and should be supplemented by further in silico analysis, as well as in vitro and/or in vivo mutation modeling prior to final determination of pathogenicity. Further validation of the pipeline will be needed prior to public release.

The current study illustrates the potential of next‐generation sequencing datasets to be mined for new discoveries long after the initial standard variant calls have been analyzed. Advances in diagnostic human genomics are now proceeding rapidly on two parallel tracks: technological and analytic. It is expected that existing next‐generation sequencing datasets will yield even more discoveries in the years to come.

## CONFLICT OF INTEREST

The authors declare no conflict of interest.
