# Supplementary figures and images for: Identification of a pathogenic mutation in ATP2A1 via in silico analysis of exome data for cryptic aberrant splice sites
Source: Mol Genet Genomic Med. 2019 Jan 28;7(3):e552. doi: 10.1002/mgg3.552 (PMC6418371; doi:10.1002/mgg3.552)

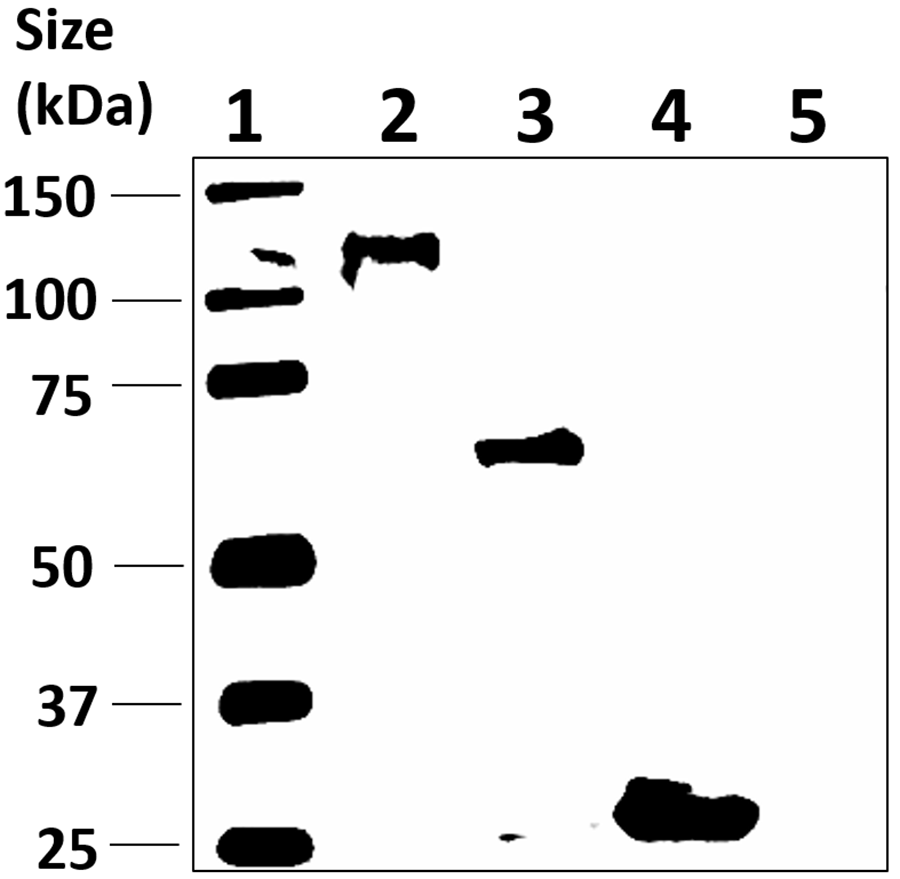

Supplement: Supplementary file 1 [file MGG3-7-na-s001.tif]
